# Paralysis of One Leg Caused by Retroversion of the Uterus

**Published:** 1868-01

**Authors:** M. M. Eaton

**Affiliations:** Peoria, Illinois


### CHICAGO MEDICAL EXAMINER.

N. S. DAVIS, M.D., EDITOR.

VOL. IX. JANUARY, 1868. NO. 1.

### Original Contributions.

### ARTICLE I.

### PARALYSIS OF ONE LEG CAUSED BY RETROVERSION OF THE UTERUS.

BY M. M. EATON, M.D., Peoria, Illinois.

Mrs. K. came under my care Feb. 3rd, 1866. She resided fifteen miles from this city, and was brought in town on a bed. Found her complaining of her left leg. There was great pain in the knee, and want of power of motion. General health tolerable; age about forty years. Native of N.H.

*History of the Case.*—About two years previous had sprained the knee in alighting from a carriage; some inflammation of knee followed, partially recovered in a month, when she fell down stairs—knee then became worse, paralysis of leg came on, and she was treated by several good physicians, when the inflammation subsided; but the want of motion and pain continued. She was placed in a "water cure establishment" for three months, became worse in general health, no improvement in leg, was taken to Chicago and treated by some one to the writer unknown, except by reputation (which is good), and was considerably improved; returned to her house without the use of her limb however. This state of affairs have continued till Feb. 3rd, 1866 (as before stated), with the additional symptoms of irritable bladder, painful menstruation, and difficult defecation. On examining the leg, I found nothing abnornal, and was led to make an examination of the uterus from the obstinacy of the case, as presented in its history, and the present symptoms, and found an enlarged uterus retroverted. There being no symptoms of pregnancy, I introduced the uterine sound the next day, and, much to my surprise, there was a discharge of a pint or more of water, after which I could discover nothing in the cavity of the uterus, and proceeded to lift the uterus to its natural position with the sound, by the aid of two fingers in the vagina, the patient being placed on her face, with the hips elevated, which I accomplished readily; kept her in this position 24 hours, and then found the uterus had contracted and remained as I had placed it. She was now placed on her side. After a week, applied the solid nitrate of silver to the internal surface of the womb, three times, at intervals of three days, when membranes, like those of a ruptured cyst, were expelled, but nothing else. I gave elix. vulerianate of ammon. to quiet the nerves, with tonics, had the affected limb rubbed freely with a stimulating liniment. She rapidly recovered; in three weeks began to walk with a cane; felt some "bearing down pain;" applied an elastic abdominal supporter, which relieved this symptom.

*April* 15*th.*—Discharged cured, since which time she has been able to do her work, dance at parties, &c. Now keeps a boarding-house, is perfectly well this 14th of November, 1867. I thought this case worthy of record, as the difficulty of the uterus had been overlooked by those who had treated her for the two years she had been unable to walk, and demonstrates that enlargement of the uterus and misplacement may effect the limbs without producing immediately any symptoms referable to the organ. The discharge of water, I suppose, was caused by the rupture of a large hydatid growth, which I inadvertantly ruptured in making an examination, expecting to find a fibrous polypus, which, by its weight, occasioned the retroversion at the time of her fall down stairs. The caustic I used to kill this growth, and prevent its refilling with water, in which I think I was successful.

Every year I am more convinced that the diseases of the uterus are too little studied by general practitioners, thereby allowing many estimable ladies to pass off the stage of usefulness to fill premature graves.

I will digress from my subject simply to add my conviction of the propriety of physicians, especially in cities and large towns, devoting especial attention to some one branch of the profession (without advertising themselves as so doing), being assured that the public will, in time, appreciate what a man is best fitted for, and patronize him accordingly. By such a course, can we not best serve our age, and the profession of medicine?

